# An Advanced Chicken Face Detection Network Based on GAN and MAE

**DOI:** 10.3390/ani12213055

**Published:** 2022-11-07

**Authors:** Xiaoxiao Ma, Xinai Lu, Yihong Huang, Xinyi Yang, Ziyin Xu, Guozhao Mo, Yufei Ren, Lin Li

**Affiliations:** 1 College of Information and Electrical Engineering, China Agricultural University, Beijing 100083, China; 2 International College Beijing, China Agricultural University, Beijing 100083, China; 3 College of Animal Science and Technology, China Agricultural University, Beijing 100083, China; 4 College of Economics and Management, China Agricultural University, Beijing 100083, China

**Keywords:** chicken face detection, generative adversarial network, masked autoencoders, deep learning, fine agriculture, intelligence agriculture

## Abstract

**Simple Summary:**

Chicken face detection is a fundamental task for accurate poultry management. Achieving satisfactory chicken face detection is necessary to implement downstream tasks, such as day-age detection, behavior recognition, and health monitoring. Nonetheless, the image dataset of the chicken face is small-scale, and there are few related studies. Moreover, chicken heads and features are smaller than other livestock, making recognition tricky. Inspired by these significances and obstacles, this paper proposes a chicken face detection network with an augmentation module. Based on the YOLOv4 backbone, our model achieved 0.91 F1, 0.84 mAP, and 37 FPS, far surpassing the two-stage RCNN and EfficientDet baselines. This model can be applied to an actual chicken coop, and its performance is adequate to conduct downstream tasks.

**Abstract:**

Achieving high-accuracy chicken face detection is a significant breakthrough for smart poultry agriculture in large-scale farming and precision management. However, the current dataset of chicken faces based on accurate data is scarce, detection models possess low accuracy and slow speed, and the related detection algorithm is ineffective for small object detection. To tackle these problems, an object detection network based on GAN-MAE (generative adversarial network-masked autoencoders) data augmentation is proposed in this paper for detecting chickens of different ages. First, the images were generated using GAN and MAE to augment the dataset. Afterward, CSPDarknet53 was used as the backbone network to enhance the receptive field in the object detection network to detect different sizes of objects in the same image. The 128×128 feature map output was added to three feature map outputs of this paper, thus changing the feature map output of eightfold downsampling to fourfold downsampling, which provided smaller object features for subsequent feature fusion. Secondly, the feature fusion module was improved based on the idea of dense connection. Then the module achieved feature reuse so that the YOLO head classifier could combine features from different levels of feature layers to capture greater classification and detection results. Ultimately, the comparison experiments’ outcomes showed that the mAP (mean average Precision) of the suggested method was up to 0.84, which was 29.2% higher than other networks’, and the detection speed was the same, up to 37 frames per second. Better detection accuracy can be obtained while meeting the actual scenario detection requirements. Additionally, an end-to-end web system was designed to apply the algorithm to practical applications.

## 1. Introduction

Farm intelligence has become an inevitable choice with the development of livestock farming in the direction of large-scale farming and precise management. This requires precise detection of individual livestock in the breeding process. Identifying and managing single individual poultry is crucial for many subsequent breeding and conservation tasks, such as tracking growth stages, detecting body condition score (BCS) [1,2], and adjusting breeding programs. The lack of monitoring of individual growth stages can lead to poor healthcare, misjudgment of heat detection, and delayed reproduction, resulting in lower production, reduced health, and even animal loss. In this study, we research the face detection of chickens.

The growth stages of a chicken can be classified by its day-old age [3]; Table 1 exhibits the details.

Particular characters of chicken are typically used as the metrics for determining the living stages. Specifically, the following: (1) Beak. The beak of a chick is sharp and thin, as is the beak angle. Adult chickens forage outdoors for a long time; after eight months, their beaks are thick and short, the end becomes hard and smooth, and the two corners of the mouth are wide and rough. (2) Nasal tumor. The nasal tumors of chicks are light red, and those of two-year-old or older chickens are light pink, large, soft, and moist. The nasal tumors of four- or five-year-old chickens are pink and rough. When judging empirically, the characteristics of the toes and feathers are also referred to. (3) Toes. Chicks have soft, tiny scales on their feet, which are bright red. The feet of adult chickens are stout and dark red, with thick, hard scales. Their toenails are hard and curved. (4) Feathers. Chicken wings can indicate the child chickens’ month-age [4]. Nevertheless, these visual methods can merely classify chickens roughly, instead of accurately determining their exact age and growth stages.

At present, traditional farms generally rely on manual records and judgments for the differentiation of chick growth stages, which can cause a large amount of labor input. Moreover, the workforce is, to some extent, based on personal practical experience, which is also generally inefficient and error-prone. In addition, some farms will use tags, spraying, and other invasive physical methods to mark the chick, so as to facilitate the subsequent record and identification management. This kind of invasive marking method will likely cause the chick to be sick, pecking tags, infected with diseases, and showing stereotyped behavior. This is not in line with animal protectionism. Noninvasive biometric methods have significant advantages over traditional methods, both in terms of cost and security. In the field of livestock individual identification and growth stage detection, increasingly more researchers are using deep learning methods. Deep-learning-based individual detection techniques have higher accuracy and robustness than traditional individual detection techniques. Moreover, detection by capturing the images of livestock does not cause harm to them. Therefore, this paper adopted a noninvasive biometric detection method and studied deep-learning-based chick face detection.

The performance of deep-learning-based methods has been optimized and improved in recent years, enhancing the implementation ability of noninvasive livestock detection management. This has been confirmed by several researchers’ projects and research results. Liang Han et al. [5] introduced a livestock detection dataset in aerial images and presented a detection algorithm to count the amount of livestock on the grassland. They first adopted a modified U-net to segment aerial images, and then obtained regions of interest (ROIs). Afterward, a Google Inception-v4 net was used to classify each ROI so as to detect objects precisely. Liyao Yao et al. [6] presented a cow face detection framework incorporating Faster R-CNN (region-based convolutional neural network) and PANSNet-5 models for detecting and recognizing cow faces. This combination successfully enhanced the recognition capability, reaching 98.3% detection accuracy and 94.1% recognition accuracy. The experiment was based on their released large-scale cow detection–recognition dataset. To track the birds’ migration movement, an automated bird-counting model [7] was built to count the number of birds in captured digital images. The RPN (Region Proposal Network) was used to select anchors with the highest likelihood of containing regions of interest. Then, the highest ones would be fed into the subsequent Fast R-CNN. The Fast R-CNN detector ultimately returns the binary label for birds’ existence and their bounding box coordinates.

Although these years have witnessed the boom of computer vision implementations in livestock detection, the applications in the field of this paper’s research are still limited—the computer-vision-based detection and artificial intelligence management for chickens are rarely seen, and concerning technologies and research data are comparatively not up to date, though chickens are one of the most common and traditional poultry. Among few pieces of research about the classification of the growth stages of chicks, one outstanding achievement is that Yufei Ren et al. [3] presented an attention encoder to find chicken face features. They implemented this structure in different mainstream CNN (convolution neural networks) to search for the most excellent network for this task. The ResNet-50 based on the attention encoder achieved 95.2% accuracy as the best. Nonetheless, the chicken face images in their dataset are with simple and ideal white background, and the chicken day ages are merely from 1 to 32 days. The situations of the subsequent days are unknown. Another excellent design is from Hung-Wei Liu and Hao-Ting Lin et al. [8], who designed a dead chicken removal system that could detect the dead chicken and sweep it with the robotic arm. They adopted a YOLOv4 deep learning algorithm to implement the detection, with the Precision reaching 95.24%, accuracy achieving 97.5%, and Recall reaching 100%. Moreover, current detection models are not ideal for individual recognition and day-old age detection, because chicken face detection tasks contain multiple objects under complicated scenarios and objects’ occlusion. Chicken faces are smaller than other livestock, and day-old age individuals have inconspicuous differences. These traits require the detection model to be capable of processing high-density objects with complex backgrounds, and the accuracy of detecting tiny features must be advanced. Some detection methods even require external devices to capture information about the livestock.

Hence, given the research contributions of the previous scientists and the lack of extant methods, this paper proposes an advanced chicken face detection network based on GAN (generative adversarial network) and MAE (masked autoencoder) modules, for detecting chicken faces from diverse growth stages.

The contributions of this paper are the following:

1. Using GAN and MAE models to augment the small volume of data in the dataset.
2. We used multiple data enhancement methods to balance the dataset.
3. We added 128×128 feature map outcomes to three feature map results, changing the downsampling of the feature map outputs to fourfold, which provides more minor object features for subsequent feature fusion.
4. We opted for the idea of dense connectivity, improving the feature fusion module to reuse features. The YOLO head classifier responsible for object detection can combine features from different levels of feature layers to obtain better object detection and classification results.
5. We applied our model equipped with cameras and edge servers for a specific chicken coop.
6. Using growth stage detection technology in livestock farms can improve the accuracy and efficiency of supervision, increasing productivity and profitability. At the same time, it reduces farming costs while following a humane and ethical approach to animal protection.

The subsequent sections of this paper are as follows: Section 2 introduces the development of object detection. Section 3 demonstrates the dataset that we used and the preprocessing methods and illustrates all the methods that we employed. Section 4 provides the validation results by conducting ablation experiments and introduces how to apply this method in practical production. Section 5 summarizes the whole work.

## 2. Related Work

The core problem of machine vision is to parse information from images that computers can understand [9,10,11]. Due to data volume accumulation, computational power advances, and their powerful representation capabilities [12], deep learning models have become a popular research field in machine vision.

Image analysis has three primary categories [13,14,15], depending on the requirements of subsequent tasks:

1. Classification.
  The classification task structures an image into a certain category of information, describing the image with a predefined category or instance identification code. This task is the most straightforward and primary image understanding task, and is the first one where deep learning models have broken out and achieved large-scale applications. Among all the excellent classification methods, ImageNet [16,17] is the most authoritative set of reviews. The annual ImageNet Large-Scale Visual Recognition Challenge (ILSVRC) has spawned many excellent deep network structures [18,19], which provide the foundation for other tasks. In the application domain, face recognition [20,21], scenes, etc., can be classified as classification tasks.
2. Object detection [22,23].
  The classification task focuses on the overall image and describes the whole picture’s content. Detection [24,25,26], however, focuses on a particular target and requires both class and location information for this target [27]. In contrast to classification, object detection provides an understanding of the image’s foreground and background. We need to divide the object of interest from the background and determine the object’s description (category and location). Object detection is a fundamental task in computer vision. Image segmentation, object tracking, and keypoint detection rely on object detection.
3. Image segmentation [28,29].
  Image segmentation includes semantic segmentation, instance segmentation, and panoptic segmentation [30,31]. Semantic segmentation [32,33] segments all objects in an image (including the background), but cannot distinguish between different individuals for the same category. Instance segmentation [34] is an extension of the detection task, which needs to describe the object’s contour (more detailed than the detection frame). Panoptic segmentation [35] is based on instance segmentation and can segment the background objects. Segmentation is a pixel-level description of an image that gives significance to each pixel class (instance) and is suitable for scenarios that require a high level of understanding, such as the segmentation of roads and non-roads for unmanned vehicles.

This paper researched chick face detection, which belongs to the mid-level processing of image analysis problems. Zhengxia Zou et al. reviewed the evolution of object detection over a 20-year period [36], including milestone detectors, datasets, metrics, and the development of significant techniques. They divided the 20-year development process of object detection into two phases, bounded by 2014: the traditional object detection period and the deep-learning-based object detection period.

Traditional object detection has high false positives for template matching, poor algorithm adaptability, practical problems that can be solved, and colossal development and maintenance costs. Its development is limited by two factors: no practical method of image representation and limited computational resources. Therefore, most of the traditional object detection algorithms are based on handcrafted features, and various acceleration techniques must be designed to reduce the dependence on computational resources. The main milestones in this cold weapon era are the following: (1) Object detection based on sliding windows [37]. This local image evaluation method detects a specific object in an image, such as birds. However, this method involves classifying and discriminating thousands of windows of different positions and sizes one by one, which consumes a lot of computational resources and makes it tricky to achieve real-time detection. (2) VJ Detector (Viola–Jones) in 2001 [38]. This is the first real-time image-based face detector based on sliding windows. This detector represents the image as integral and can quickly compute Haar-like features. (3) HOG (histogram of oriented gradients) detector in 2005 [39]. The gradient features of the whole image are extracted, and the HOG features are formed by extracting and dividing the detection window and splicing the histogram features to finally achieve human detection. (4) DPM (deformable part model) [40] is a component-based detection feature and algorithm derived from HOG, which extracts more discriminative features on the basis of HOG features.

After 2014, called the “era of the beauty of GPU (graphics processing unit)”, deep-learning-based detectors are divided into CNN-based two-stage detectors (R-CNN, SPP-NET, Fast R-CNN, Faster R-CNN) and CNN-based one-stage detectors (YOLO, SSD). Until today, researchers have designed network structures, optimized methods, and loss functions to improve the effectiveness of model detection. The year 2014 saw the introduction of R-CNN [41], the pioneer of deep learning in object detection, proposing classification and localization based on candidate frames. However, R-CNN severely affects the quality of CNN extracted features when unifying the region proposal size, and also spends a lot of computation time and storage space when extracting the region proposal features. Therefore, the SPP-NET (spatial pyramid pooling network) was introduced to extract the features of the whole image at one time, which can handle region proposals of arbitrary size, improving the quality of the features extracted by CNN and enhancing the robustness. In 2015, Ross et al. proposed Fast R-CNN [42], which optimized the loss function and changed the pooling strategy to accelerate CNN’s training and prediction significantly. However, its extraction of region proposals took a longer time. In the same year, Faster R-CNN [43] was introduced, which used RPN (Region Proposal Network) [43] to replace the selective search region proposal extraction method (which had been used in the previous R-CNN family of networks) and greatly improved the detection speed. In 2016, the “cost-effective choice” YOLO (You Only Look Once) [44] was proposed, and SSD (single-shot multiBox detector) was introduced. In 2017, FPNs (feature pyramid networks) [45] and Mask R-CNN [46] were proposed. In 2018, IoU-Net [47] was proposed, and in 2019, GIoU-Net [48] was proposed. So far, object detection methods have been refined, improved, and broken through.

Although object detection and deep learning methods are changing rapidly, it does not mean that the age-old methods are not relevant today. The most typical example is AlexNet [18], which emerged in 2012. Alex used two GPUs for parallel computing, and proposed many preprocessing methods for image data augmentation. He also introduced the first ReLu nonlinear activation function, Dropout, and LRN (local response normalization) techniques, which significantly improved the training rate of the network and reduced the error rate, opening the way for later generations to explore object detection methods. The foundation of the method and ideas was laid.

## 3. Materials and Methods

### 3.1. Dataset Analysis

In this paper, the dataset was acquired by both manual means and camera. Among them, the manual acquisition was taken by the photographer, who is responsible for taking the appropriate images, and a camera made the camera acquisition. Because the camera is at a fixed position and fixed angle, we only kept the images in the central area of the camera (the edge distortion is severe, and human eyes cannot recognize it, so it cannot be labeled with ground truth). The reason for using these two approaches is that they basically cover most of the applicable scenarios, and thus we can ensure that the model trained in this way can be applied to most of the farms.

**Figure 1:**
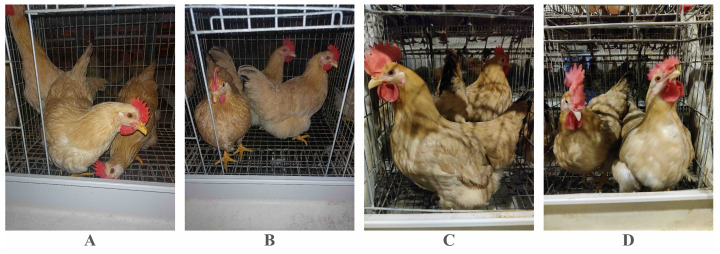
Exhibition of the chicken dataset. (**A**–**D**) represent day-old chickens from different sub-datasets.

The Guangdong Academy of Agricultural Sciences, China, manually collected the dataset adopted in this paper. Researchers used a Canon 5D digital camera to capture these images on 20 March 2022. Figure 1 and Table 2 [3] display details of this dataset, and each image’s resolution is 6720×4480.

### 3.2. Data Augmentation

**Figure 2:**
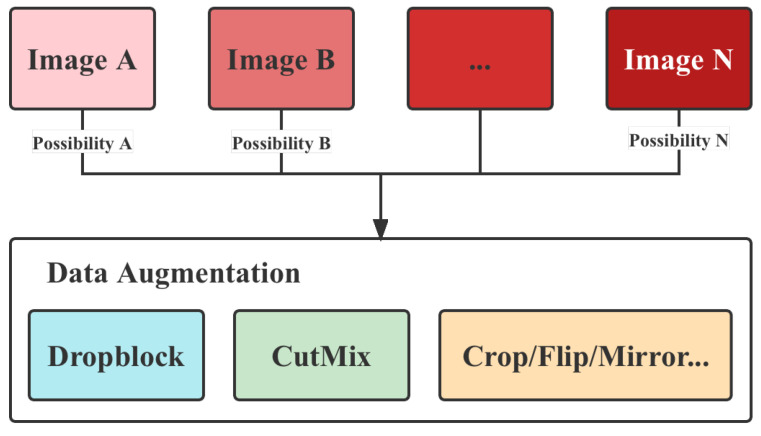
Illustration of the data augmentation process.

As can be seen from Table 2, the dataset used in this paper is not balanced. The imbalance will lead the model to favor features from classes with numerous learning amounts, such as 121–150 days of age, and ignore learning features from weak classes, such as 31–60 days of age. To address this issue, we augmented the dataset with data augmentation methods. Rather than treating all classes equally, the augmentation process involves multiple augmentations for weak classes and small probability augmentations for strong classes (i.e., only a certain probability of being augmented). This process would balance the number of different types of images in the training set. Specifically, the multiplicity of enhancements for a class (the probability of augmentation for strong classes) is inversely proportional to the number of training sets for that class, i.e., the smaller the number, the greater the enhancement, as shown in Figure 2.

#### 3.2.1. CutMix

The augmentation method of CutMix [49] is to perform the operation on a pair of pictures by randomly generating a cropping box and cropping off the corresponding position of the *A* image. Then, we use the ROI of the corresponding position of the *B* image to place the cropped area in the *A* image to create a new sample. The ground truth label is adjusted proportionally according to the area of the patch. The weighted summation is used to solve the loss calculation.

The CutMix method is optimal compared to MixUp [50] and Cutout on the ImageNet Cls, ImageNet Loc, and Pascal VOC Det datasets. Where Mixup is a direct summation of two images, it is challenging for the model to learn the exact feature map response distribution. Cutout directly erases a region of the image, which forces the model not to be overly confident in a particular feature when performing classification. Nonetheless, a part of the image is filled with useless information. The CutMix method will cut a portion of an image and paste it onto another image, making it easier for the model to distinguish between dissimilarities.

#### 3.2.2. DropBlock Regularization

Regularization techniques help to avoid the most normal issue faced by data science professionals, i.e., overfitting. Several methods have been proposed for regularization, such as Dropout [51], Early Stopping, L1 and L2 regularization, and data augmentation. In this paper, we used the DropBlock regularization.

**Figure 3:**
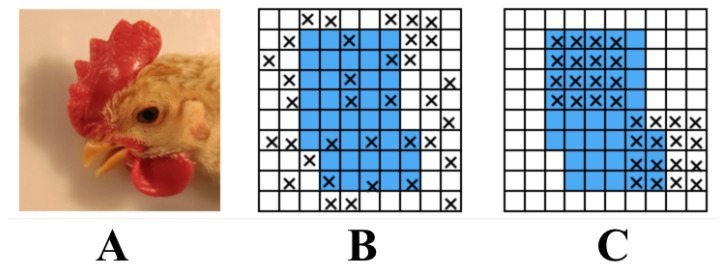
The effect of DropBlock regularization. (**A**) is the original image and the blue parts in (**B**,**C**) represent the activation units containing semantic information, which cannot be removed. The black cross parts are marginal features of chicken faces.

The DropBlock method was introduced to overcome the main drawback of the Dropout method—randomly discarding features. The Dropout method proved an effective strategy for fully connected networks but was ineffective in feature-space-dependent convolutional layers. The DropBlock technique discards features in adjacent correlation regions called blocks. This achieves the goal of generating simpler models, but also reduces overfitting by introducing the concept of learning some of the network weights in each training iteration and compensating for the weight matrix. The effect of DropBlock is shown in Figure 3.

#### 3.2.3. Modified Label Smoothing

For classification tasks, especially multiclassification tasks, vectors are often converted into one-hot vectors. Nevertheless, the one-hot type brings problems: we must fit the true probability with the predicted probability for the loss function. Fitting the true probability function of one-hot brings two problems:

1. Inability to guarantee the model’s generalizability, which tends to cause overfitting.
2. The probabilities of one and zero encourage the widest possible gap between the category to which they belong and the other categories, which is difficult to accommodate as known by the gradient being bounded. It can cause the model to place too much confidence in the predicted categories.

Having 100% confidence in the prediction might indicate that the model memorizes data rather than learns them. Label smoothing changes the target’s upper limit of the prediction to a lower value, say 0.9. It will utilize this value instead of 1.0 to calculate the loss. This concept mitigates overfitting. To be clear, this smoothing somewhat reduces the gap between minimum and maximum in the label. Label smoothing reduces overfitting. Therefore, adjusting the label appropriately so that the extreme values at both ends come together towards the middle can increase the generalization performance.

### 3.3. Augmentation Networks

#### 3.3.1. Generative Adversarial Networks

Generative adversarial networks (GANs) are among the most promising approaches for unsupervised learning on complex distributions. Models learn by playing each other through (at least) two framework modules, the generative models and discriminative models, to produce fairly good outputs.

This structure is used in [33,52,53,54,55,56] to augment the image dataset, so in this paper we also used this model for dataset augmentation. The specific steps are:

1. Determine the target identification object.
2. Make a dataset *M* of target objects (the dataset *M* is relatively small).
3. Collect a large amount of public data *N* of similar objects in other environments through methods such as Baidu platform.
4. Model training. Train *N* to a data distribution $N{}^{\prime }$ of similar style to *M* by GANs model.
5. Add $N{}^{\prime }$ to *M*.

#### 3.3.2. Masked Autoencoders

Masked autoencoders (MAEs) [57] first apply a random mask to the input image patch, then reconstruct the missing pixels. MAE is based on an asymmetric encoder–decoder structure. (1) The encoder operates only on a subset of the unmasked patch part. (2) Then, a lightweight decoder reconstructs the image from the hidden space and mask token. In this paper, 75% of the chicken face images are masked for reconstruction, and we still obtained meaningful self-supervised results.

In vision tasks, the decoder is responsible for recovering the input hidden space representation back to the pixel level. Its output has a lower semantic level than the general recognition task. In contrast, in language processing tasks, the decoder needs to predict the missing words, which are rich in semantic information. Although the decoder is not very important in BERT (bidirectional encoder representation from transformers), and the final output is obtained by only one layer of MLP (multilayer perceptron), the decoder is crucial in the data amount task, which determines the level of semantic level that can be represented.

This paper proposes a plus-single, efficient, and scalable MAE based on the above analysis to learn visual representations. MAE will apply a random mask to the input image, and then reconstruct the missing parts in the pixel space.

**Figure 4:**
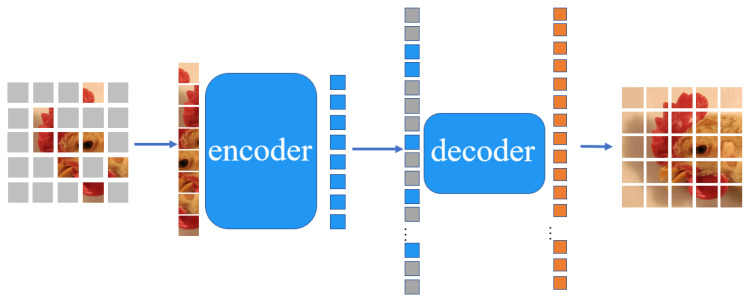
The asymmetric encoder–decoder structure of MAE. The image is masked, and the unmasked patches are input into the encoder. The encoder codes them into more patches and delivers them to the decoder to reconstruct a whole unmasked image.

MAE is an asymmetric encoder–decoder structure, where the encoder only operates on unmasked patches, while the decoder is responsible for reconstructing the image from the hidden space in combination with a mask token, as shown in Figure 4.

**Figure 5:**
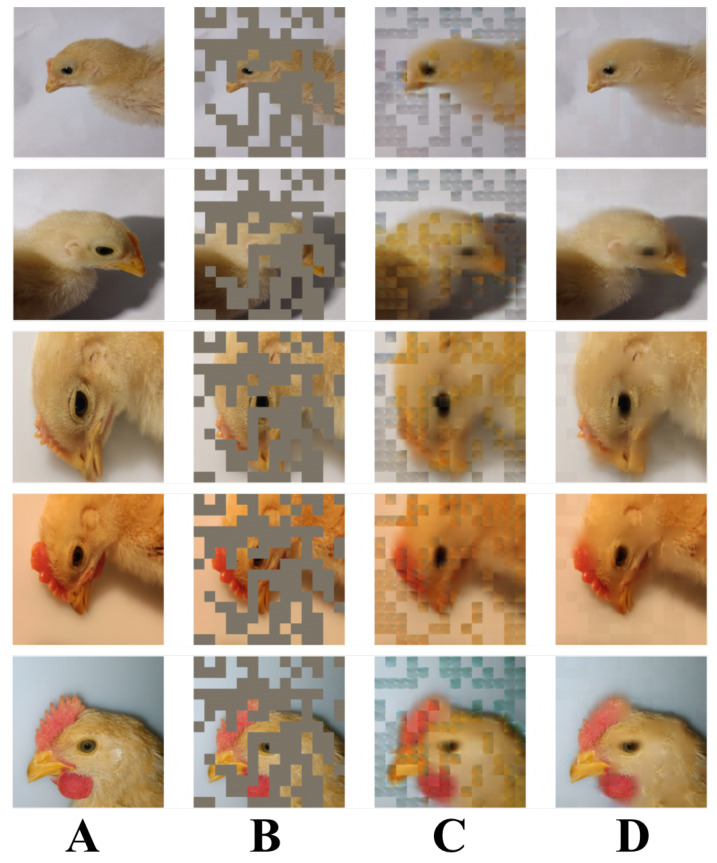
The data-generating effect of MAE. (**A**) Original image series; (**B**) masked image series; (**C**) half-reconstructed image series; (**D**) reconstructed unmasked image series.

MAE randomly masks a large fraction of the patches (e.g., 75%) during pretraining. The encoder then processes the unmasked patches. The decoder inputs all unmasked patches and mask tokens, which are used to reconstruct the input image. After pretraining, the decoder is discarded, then only the encoder is used for the unprocessed images, and the learned visual representations are used for the recognition task. Figure 5 illustrates the effect of using MAE to generate the dataset.

### 3.4. Core Detection Network

This paper adopted the YOLOv4 detection network to conduct a proper innovative algorithm to achieve a perfect balance of speed and accuracy. These include weighted residual connections (WRCs), cross-stage-partial connections (CSPs), cross-mini-batch normalizations (CmBNs), self-adversarial training (SAT), improved Mish activation function, mosaic data augmentation, CmBN, DropBlock regularization, CIoU loss, etc. [58].

Undeniably, YOLO series networks balance the detection accuracy and inference speed, which is somehow the best cost-performance choice. Meanwhile, a specific YOLO network was improved for a particular application among the YOLO series networks. We will discuss the reason why we employed YOLOv4 as the core detection network in Section 4.1 by conducting contrast experiments with RCNN series, SDD, and other YOLO series networks.

#### 3.4.1. CSPDarknet53

**Figure 6:**
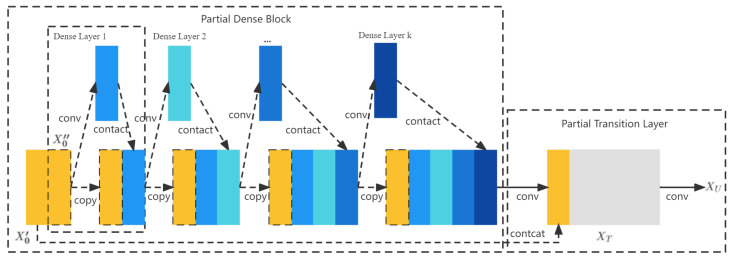
The structure of CSPDarknet53, in which different color blocks represent different functional layers; orange represents the input layer, blue represents the convolution layer, and the darker color represents the higher convolution level.

In YOLOv3, the feature extraction network uses Darknet53, while in YOLOv4, a little improvement is made to Darknet53 by borrowing CSPNet. The full name of CSPNet is cross-stage-partial network, that is, cross-stage local networks. CSPNet solves network optimization’s gradient information duplication problem in other extensive CNN frameworks, integrating the gradient changes into the feature map from the start to end. Hence, it reduces the number of parameters and FLOPS (floating-point operations per second) values of the model, ensuring both the speed and accuracy of inference and reducing the model size. The structure is shown in Figure 6.

CSPNet is actually based on the idea of DenseNet, which copies the feature mapping map of the base layer and sends a copy to the subsequent stage through the dense block, thus separating the feature mapping map in the base layer. This can effectively alleviate the gradient disappearance problem (it is challenging to backpropagate the lost signal through a profound network), support feature propagation, and encourage the network to reuse features, thus decreasing the number of network parameters. The CSPNet idea can be combined with ResNet, ResNeXt, and DenseNet. Currently, there are mainly two kinds of retrofitting backbone networks, CSPResNext50 and CSPDarknet53.

A pleasing classification effect of a model does not necessarily imply that its detection effect is excellent. It has to consider the balance of several aspects: the resolution of the input network, the number of convolutional layers, the number of parameters, and output dimensionality. Additionally, the following points are required for an excellent detector:

1. Larger network input resolution—for detecting small objects.
2. Deeper network layers—able to cover a larger receptive field area.
3. More parameters—better detection of different-sized objects within the same image.

**Figure 7:**
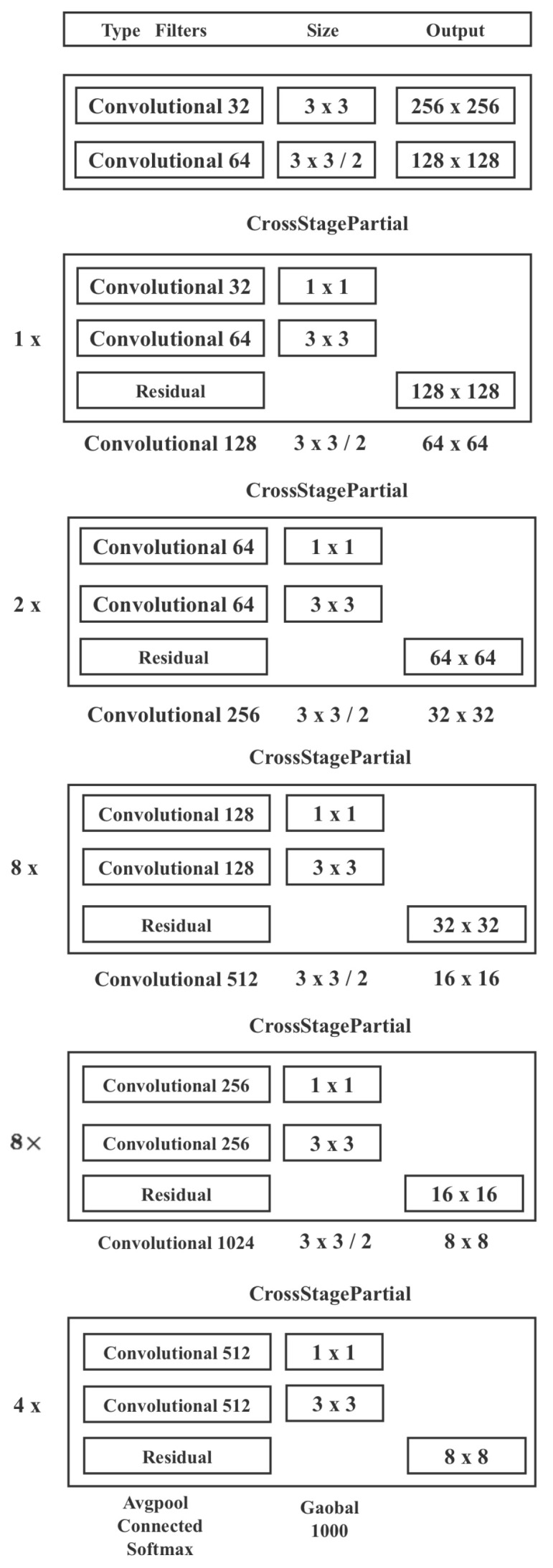
The ultimate structure of CSPDarknet53.

The ultimate structure of CSPDarknet53 is illustrated in Figure 7.

#### 3.4.2. Spatial Pyramid Pooling Structure

**Figure 8:**
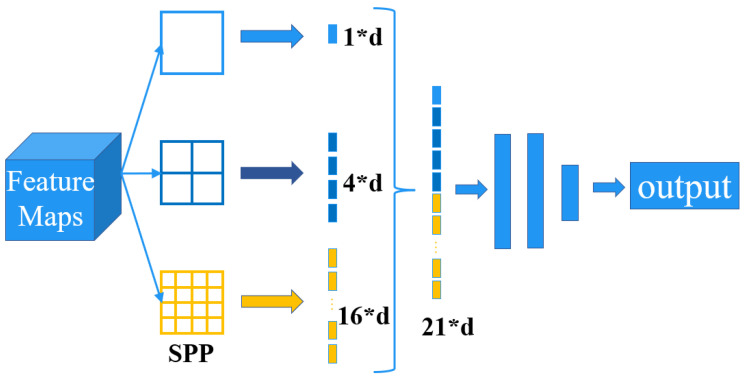
The structure of the SPP-Net. Example of constructing a three-level pyramid.

The spatial pyramid pooling network (SPP-Net) [59] is mainly used to tackle how different-sized feature maps enter the fully connected layer. The structure of the SPP-Net is shown in Figure 8.

The structure allows direct pooling of arbitrary-sized feature maps to obtain a fixed number of features with fixed dimensions.

#### 3.4.3. Path Aggregation Network Structure

**Figure 9:**
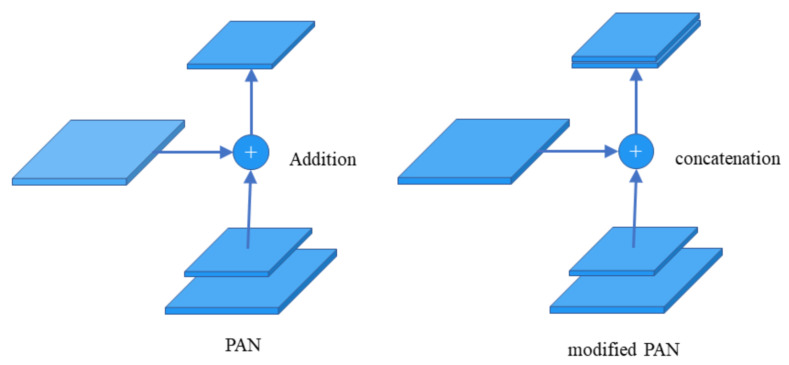
The difference between addition and concatenation fusion methods.

Using PANet (path aggregation network) [60] instead of FPN for parameter aggregation can be applied to different levels of object detection. The method used for fusion in PANet is addition, and in this paper, the method of fusion is changed from addition to concatenation. The difference between the two fusion methods is shown in Figure 9.

### 3.5. Experiments

#### 3.5.1. Experiment Settings

To conduct experiments, we used a desktop computer with an Nvidia RTX3080 GPU and a Core i9-10900k CPU, the experiments were run on Windows 10 and the programming language was Python 3.9; the model was implemented using the PyTorch 1.8 framework. The number of learning epochs was 150, and the optimization function was the stochastic gradient descent algorithm, with the initial learning rate being $1\times 10^{-5}$.

#### 3.5.2. Model Evaluation Metrics

We utilized Recall (R) and Precision (P) as the evaluation metrics to confirm the model’s efficacy in detecting chicken faces. Table 3 displays the assessment criteria that were applied to the object detection process.

Here, TP represents chicken face numbers detected as valuable positive objects and contained; FP stands for chicken numbers detected as false-positive objects and not contained; FN stands for the amount of false-negative objects not detected and contained; TN represents chicken face amounts that are true objects not detected and not contained.

Precision (*P*) denotes the percentage of detected objects included in images of detected objects and is formulized by Equation (1):

(1) $$Precision=\frac{TP}{TP+FP}$$

Recall (*R*) indicates the percentage of images containing truly detected objects and is formulized by Equation (2):

(2) $$Recall=\frac{TP}{TP+FN}$$

F1 balances Precision and Recall and is expressed by Equation (3):

(3) $$F1=\frac{2\times Precision\times Recall}{Precision+Recall}$$

Although the AP computation varies slightly depending on the dataset, the PR curve’s area is generally calculated to calculate the AP value. VOC2007’s computation first smooths the curve and moves the greatest Precision value to the right of each point to create a straight line. COCO utilized 101 interpolation points to compute AP more accurately. Moreover, it calculated AP in term of diverse intersection over union (IoU) thresholds. AP calculation is merely for a single class. After obtaining the AP value, the mAP is comparatively straightforward: calculating AP for all classes and then averaging. The following is the calculation, Equation (4):

(4) $$mAP=\frac{\sum_{i=1}^{num\_class}AP_{i}}{num\_class}$$

This paper selected F1, mAP, and FPS (frames per second) as evaluation metrics.

## 4. Results and Discussion

### 4.1. Validation Results

Table 4 gives all conducted test outcomes, including one-stage network representatives: YOLO series, SSD, efficientDet [25], and two-stage network representatives: Mask RCNN [46] and Faster R-CNN.

Experimental results from Table 4 indicated that the proposed model has a comparatively fast inference speed, with an FPS speed of 37. Our model’s inference performance surpassed the two-stage networks and the EfficientDet. Nevertheless, YOLO series and SSD models have a better inference performance. The highest value, 58 FPS, was achieved by YOLOv5. The F1 and mAP of Faster R-CNN are 0.71 and 0.65, and its performance is the worst among all models. The F1 and mAP of YOLOv5 surpassed Faster RCNN, Mask RCNN, and SDD. Additionally, YOLOv5 performed best among all YOLO series in the experiment. Though EfficientDet has a better F1 value than other contrast networks, its mAP metric is merely higher than that of the Faster RCNN. This may be because EfficientDet has an attention extraction module, which can facilitate a higher F1 metric. Conclusively, YOLOv3 and YOLOv5 worked best in the experiment. Our model reached 0.91 F1 and 0.84 mAP, respectively, well over the other contrast models.

### 4.2. Detection Outcomes

**Figure 10:**
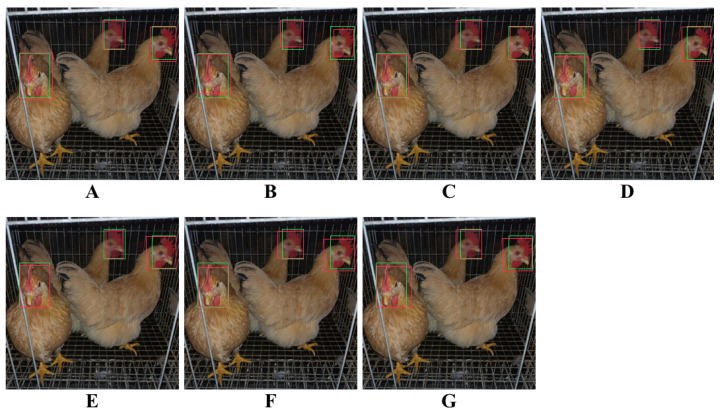
Illustration of detection outcomes from different detection models. (**A**) This study, (**B**) Faster RCNN, (**C**) Mask RCNN, (**D**) YOLOv3, (**E**) YOLOv5, (**F**) SSD, and (**G**) EfficientDet.

We extracted several images from the test set images for further comparison. These images possess multiple detection scenes in our dataset, such as images with significant differences in day-age, and scenes with large differences in illuminances and backgrounds in the images. Figure 10 illustrates the detection outcomes from different object detection networks. Red boxes represent ground truths, and the green boxes denote the predicted bounding box generated by networks.

Figure 10 shows that Faster-RCNN did not perform pleasingly in these images, while SSD series, EfficientDet, and YOLO series performed comparatively well and could detect the chicken face region accurately. Nevertheless, when the percentage of detected images is too low, i.e., the granularity of the region to be detected is too small, all models’ performances degrade. This situation might be related with the attention extraction module in these networks.

Our model exceeded other models in these experimental detection outcomes, though there is still room for improvement. Our model performed agreeably even when detecting images with high-density chicken faces. Since our model employs fewer parameters and has lower complexity, it is possible to deploy the algorithm to low-cost hardware.

#### Application for Downstream Tasks

**Figure 11:**
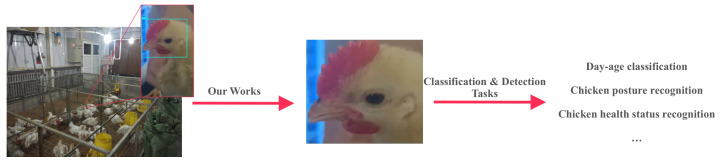
Schematic diagram of the upstream and downstream task network based on the model in this paper.

In an actual chicken coop scenario, we need to identify the behavior of each chicken in the camera, such as sleeping and drinking; we need to detect whether the chicken is sick or not. Moreover, whether the chicken has its eyes open is one of the essential criteria (if other downstream tasks need to be performed using the chicken’s facial features, we can add them). Therefore, prescreening the chicken’s facial region in complex scenarios will play an essential role in the subsequent tasks. Figure 11 shows the overall application flow.

**Figure 12:**
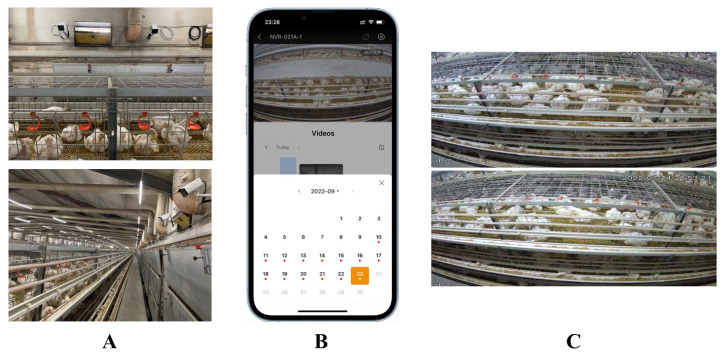
Intelligent system of chicken coop based on the model of this paper. (**A**): HD camera in the chicken coop; (**B**): app for accessing video stream; (**C**): video stream display.

Based on this, we designed the chicken coop intelligence system shown in Figure 12 for the model proposed in this paper to facilitate the use of this model.

As can be seen, the hardware part of the system consists of a 4k camera deployed in the chicken coop for capturing individual chickens, and an edge server deployed in the edge scenario for storing the captured videos, which can be accessed in real time through an app. On the server side, our algorithm can automatically annotate the captured video with frames and crop out the chicken face region to prepare for the downstream classification and detection task.

### 4.3. Discussion

#### 4.3.1. Ablation Experiments for Various Data Augmentation Methods

In order to investigate whether the GAN and MAE models used in this paper can effectively improve the model’s performance, we conducted ablation experiments, as shown in Table 5.

The table shows that using different GAN models for dataset generation can improve the model’s performance to different degrees. However, all GAN models can improve the mAP of the model. This may be because different GAN models have different implementation details, and the quality of the generated dataset is not uniform. Although the quality of the generated dataset may be high or low, it is still similar to the original dataset, belonging to the approximate distribution. Since the underlying condition for each training batch of deep learning is independent identically distribution, all GAN models can improve the model’s performance when the distribution approximation condition is satisfied.

Among all the GAN implementations, DCGAN achieved the best boosting effect. This is because the generators and discriminators in DCGAN were implemented using deep convolutional networks instead of MLPs in the original GAN model. Therefore, GAN has a stronger representation and learning capability and generates a better dataset, i.e., the distribution is closer to the original dataset.

Moreover, the effect of using the MAE method to conduct data augmentation is superior to all the other GAN augmentation methods. This is because MAE is essentially a learning process based on the original dataset, which borrows the concept of contextual relationship from natural language processing. MAE learns the contextual relationship between different blocks in the dataset, and then learns how to use the known blocks to reason about the generated blocks. Therefore, the generated distribution must be closer to the original distribution than GAN, i.e., the generated dataset is of higher quality. The fundamental reason is that MAE is generated on a masked dataset, while GAN is generated from a random distribution. Finally, the best detection results can be obtained by augmenting the original dataset with MAE and GAN.

#### 4.3.2. Ablation Experiments for Various Data Augmentation Methods

In order to investigate whether the various data augmentations used in this paper can effectively enhance the model’s performance, we conducted ablation experiments, as shown in Table 6.

Table 6 indicates that the CutMix method is the most effective for data augmentation. In comparison, the DropBlock regularization method has the worst F1 and mAP, which does not have a more positive effect among these methods. However, each enhancement method can improve our model’s mAP.

#### 4.3.3. Future Work

The chicken face is an essential feature in identifying chickens. To recognize the chickens’ age, we already performed day-age recognition on chicken face data [3]. However, in a natural chicken farm environment, as in Figure 11 and Figure 12, we cannot obtain chicken face images directly. Hence, this paper focused on detecting and cropping the chicken face region from the images collected in the chicken farm. Determining how to apply the obtained chicken face parts to downstream tasks, as mentioned in Section 4.2, such as chicken day-old age, behavior recognition, or chicken disease recognition, will be the future research direction of the authors.

## 5. Conclusions

Refined and intelligent management of livestock farming is becoming increasingly important in agricultural production. The facial recognition of individual poultry according to day age is the basis for carrying out downstream production tasks. Considering the requirement and traditional difficulties in poultry detection, we proposed a deep network model that can accurately detect chicken faces with the following innovations:

1. We augmented the limited-scale dataset by employing the GAN and MAE models. Compared with the baseline method without data augmentation, our GAN-MAE augmentation method increased the F1 and mAP from 0.87 and 0.75 to 0.91 and 0.84, respectively.
2. We solved the imbalance between different classes of the dataset using multiple data enhancement methods.
3. We added 128×128 feature map outputs to three feature map outputs of this algorithm, thus changing the eightfold downsampling of the feature map outputs to fourfold downsampling, which provides more small object features for subsequent feature fusion.
4. The feature fusion module was improved based on the idea of dense connectivity to achieve feature reuse. The YOLO head classifier responsible for object detection can combine features from different levels of feature layers to obtain better object detection and classification results.
5. Our model achieved the most exceptional performance in the contrast experiments, with 0.91 F1, 0.84 mAP, and 37 FPS, which are well over those of the two-stage models and EfficientDet.
6. We deployed our camera and edge server for a specific chicken coop, and applied our model.

Although our research obtained the aforementioned breakthroughs and achievements, there are still some limitations. The day-old age interval of our dataset is 30 days, which is not refined enough. We just identified the face of chickens from different growth stages, but the specific day-age, behavior, and health condition cannot be classified. Our model gained the most promising F1 and mAP outcomes; however, the inference speed is inferior to YOLOv3 and YOLOv5. These interesting research areas are worthwhile to be further explored, and they will also be the future work of the authors in this paper.

## Figures and Tables

**Table 1:** Growth stages of chicken categorized by day-old ages.

Growth Stages	Living Situations
Chicks	Newborn–60 days of age.
Breeding stage	61–150 days of age.
	breeding chickens
Adult stage	151 days of age and above.
Reserved chickens	Hens that have not begun laying eggs;
	breeding roosters that have not yet been mated.

**Table 2:** Data samples’ distribution of the dataset.

Day-Old Age	Amount of Data Samples
1–30	6491
31–60	1321
61–90	1467
91–120	4287
121–150	7109
150+	6988

**Table 3:** Matrix of classification metrics.

Label/Prediction	Positive	Negative
Positive	TP	FP
Negative	FN	TN

**Table 4:** Comparison of two-stage, YOLO series, EfficientDet, SSD detection models, and ours.

Method	F1	mAP	FPS
Faster RCNN	0.71	0.65	33
Mask RCNN	0.75	0.71	29
EfficientDet	0.86	0.71	35
YOLOv3	0.85	0.73	51
YOLOv5	0.87	0.72	**58**
SSD	0.82	0.68	45
ours	**0.91**	**0.84**	37

The most excellent outcomes for each evaluation metric are shown in bold.

**Table 5:** Results of different data augmentation methods.

Method	F1	mAP
No augmentation (baseline)	0.87	0.75
GAN	0.88	0.75
DCGAN	0.89	0.78
MAE	0.91	0.81
DCGAN + MAE	0.91	0.84

**Table 6:** Results of different data augmentation methods.

Method	F1	mAP
CutMix	0.90	0.79
DropBlock	0.85	0.75
Modified label smoothing	0.87	0.76

## Data Availability

Not applicable.
